# Eye Care Service Use and Associated Health-Seeking Behaviors Among Malawian Adults: Secondary Analysis of the Malawi Fifth Integrated Household Survey 2019-2020

**DOI:** 10.2196/44381

**Published:** 2024-04-09

**Authors:** Thokozani Mzumara, Marios Kantaris, Joseph Afonne

**Affiliations:** 1 Department of Optometry, Mzuzu University, Mzuzu, Malawi; 2 Department of Ophthalmology, Mzimba North District Hospital, Ministry of Health, Mzuzu, Malawi; 3 Unicaf University, Lusaka, Zambia; 4 Health Services and Social Policy Research Centre, Nicosia, Cyprus

**Keywords:** access to health, health service utilization, eye care use, health-seeking behavior, sociodemographic determinant, visual impairment, social support, women empowerment, education, eye care, pediatric, eye, ophthalmology, visual, ECU, eye service, utilization, Malawi, empowerment, health service use, use

## Abstract

**Background:**

The use of eye care services varies among different population groups.

**Objective:**

This study aimed to assess self-reported eye care use (ECU) and associated demographic factors among Malawian adults.

**Methods:**

This study used secondary data from the Malawi Fifth Integrated Household Survey 2019-2020, a nationally representative survey. The study included 12,288 households and 27,336 individuals 15 years and older. We entered age, sex, level of education, residency (urban/rural), and chronic disease into a logistic regression model, and used a confusion matrix to predict the model’s accuracy. A *P* value <.05 was considered statistically significant.

**Results:**

About 60.6% (95% CI 60.0%-61.2%) of those with eye problems accessed formal care 2 weeks before the survey date. A logistic regression model showed that ECU was positively associated with education compared to none (odds ratio [OR] 6.6, 95% CI 5.927-7.366; *P*<.001), males compared to females (OR 1.2, 95% CI 1.104-1.290; *P*<.001), and urban residence compared to rural (OR 1.2, 95% CI 1.118-1.375; *P*<.001). ECU was negatively associated with age (OR 7, 95% CI 6.782-8.476; *P*<.001) and having chronic diseases (OR 0.6, 95% CI 0.547-0.708; *P*<.001).

**Conclusions:**

Social support, women empowerment, education, and mobile clinics are key strategic areas that would increase access to eye care in Malawi. Further studies can investigate ECU among the pediatric population.

## Introduction

Life, health, and sustainable development depend on good ocular health, yet many people cannot afford the eye care they need, which can result in blindness or visual impairment [1,2]. Approximately, 90% of the 596 million individuals with visual impairments globally in 2020 lived in low-income nations [3,4]. Access, eye care use (ECU), and poverty are all directly correlated with the prevalence of visual impairment [2]. According to the literature [5], poverty is a direct cause and effect of blindness. It affects impoverished communities in certain ways and prevents the poor from accessing eye care services because the associated expenses are too high. Socioeconomic variables including education and ethnicity are to blame for disparities in health-seeking behavior (HSB), access to services, visual impairment, and blindness [5]. Blindness exacerbates poverty, which results in diminished autonomy, position, and authority as well as decreased involvement, social isolation, and stigma. This can extend to other family members, leading to despair and decreased productivity. Beyond a lack of money, poverty also includes a loss of control over one’s life; a loss of status, authority, and prestige; fewer possibilities to engage in family- and work-related activities; stigma; and social isolation.

ECU entails using eye activity for treatment, prevention, and health promotion [6-8]. Health care use is influenced by sociocultural variables, norms, autonomy, and decision-making. Additionally, human behavior poses a challenge to cost-effective health care solutions [9]. HSB is influenced by the health belief model, driven by accessibility to care, perceived severity, susceptibility, and treatment benefits [10]. The model distinguishes potential and realized access, with predisposing factors like age, sex, and lifestyle; need factors like ill health; and enabling factors like wealth and proximity to health facilities [11]. Nevertheless, HSB is not homogeneous and relies on cognitive and noncognitive contexts [10]. The factors affecting HSB are multifaceted; hence, wide variations are common, making it a challenge to health equity and universal eye health.

Globally, ECU varies from 18% to 83% [6] and is characterized by inequalities based on sex, comorbidity, age, and education [12]. Furthermore, ECU varies between rural and urban residences [12]. Studies have found that visual problems are higher among those less educated and who live in rural areas [5]. Numerous studies have looked into the ECU pattern and the risk variables that are linked to it [6,13-18]. This article attempts to evaluate the pattern of ECU among adults in Malawi, concentrating on related demographic parameters, given that ECU varies with context. The study also looks at the decisions made by individuals who have vision issues, including their reasons for not seeking eye care.

Malawi is among the world’s poorest nations [19]. According to a recent study, Malawi must first eradicate inequality before poverty can be eliminated [20]. The results of this study can help address the systematic exclusion of disadvantaged groups and develop health systems toward universal coverage of eye health through evidence-based programs and policies to increase the use of eye care services. The attainment of universal eye health is hindered by low uptake of eye care, especially in low-income countries where access to care is restricted and patients delay seeking treatment due to financial restrictions, fear, or neglect [6,10,21].

## Methods

### Survey

This quantitative cross-sectional correlation study was conducted as a desk-based review using data obtained from the Malawi Fifth Integrated Health Survey (IHS) 2019-2020 [22]. The survey, a living standards measurement study, was implemented between April 2019 to April 2020. The survey used a nationally representative sample and was conducted across districts in Malawi.

### Sampling Procedure

The survey used a stratified 2-stage sampling procedure. The sampling frame was based on listing information and cartography from the 2018 Malawi Population and Housing Census [22], which included the three major regions, namely, the north, center, and south stratified into rural and urban areas. The urban strata included Lilongwe, Mzuzu, Blantyre, and Zomba—as such, the survey considered all other districts to be rural. The study sampled across 32 districts in the nation. The frame excluded populations in institutions such as prisons, hospitals, and military barracks. First, enumeration areas based on the 2018 census were selected within each stratum. Next, households were selected from the EA using systematic sampling. Finally, 12,288 households from 780 enumeration areas were selected with a 93% response rate. The survey interviewed all individuals 15 years and older living in the selected household.

### Sampling Weights

The sample estimates from the Fifth IHS were multiplied by sampling weights so that the findings of the study could be extrapolated to the whole population 15 years and older [22].

### Data

This review extracted age in years, sex (male/female), residence (rural/urban), highest level of education, and sampling weights from the household module of the survey. The age of participants was regrouped into young adults (15-34 years), middle-aged (35-59 years), and older adults (60 years and older). Participants’ level of education was recoded into five categories, namely, none (no education or do not know), primary (primary school leaving certificate), secondary (including A level), and tertiary (diploma, first degree, master’s degree, or PhD). In addition, the paper extracted information on whether the individual had experienced any symptoms for the previous 2 weeks (yes/no), symptom names, action taken to relieve the symptom, and having a chronic illness.

To determine ECU, we recorded the action taken to relieve the eye symptom as 1 if the individual sought care at a government or private facility including a church/mission facility, village clinic, and pharmacy store. All other options, including self-care and use of stock medicine and other nonorthodox practices, were recoded as 0.

### Analysis Strategy

The data were entered into SPSS, version 26 (IBM Corp). We analyzed descriptive statistics including mean and SD, frequency, and proportion. A graphical illustration of the results is presented in tables. Proportional data were analyzed using *χ*^2^, and we considered a *P* value <.05 as significant. The variables were entered into a binary logistic regression model using the entry method to estimate the probability of ECU occurring. The probability cutoff was set at 0.5%—probabilities greater than 0.5 were classified as ECU (event occurring); otherwise, they were classified as no ECU (event not occurring). To assess the prediction classification model, the study used the confusion matrix technique and calculated the predictive values accuracy, specificity, and sensitivity of the model/classifier.

### Ethical Considerations

The study adhered to the tenets of the Declaration of Helsinki. Explicitly, the IHS survey obtained informed consent from all participants. Nevertheless, the study did not require institutional review because it used deidentified publicly available data. We obtained permission and data sets from the World Bank [23]. The study subjects participated freely and were not compensated in any form.

## Results

### Prevalence of Eye Symptoms 2 Weeks Preceding the Survey

Of the 27,336 participants recruited, 8014 (29.3%) were males. The mean age of participants was 47.68 (SD 24.39) years. The proportion of participants with symptoms of eye problems 2 weeks before the study was highest among older adults (60 years and older; n=11,155, 40.8%). According to the region, eye symptoms were higher in the south (n=12,378, 45.3%) and lowest in the north (n=3084, 11.3%). The majority (n=23,202, 84.9%) were from rural areas. The majority of participants with eye symptoms were married (n=14,140, 51.7%; *P*<.001; Table 1).

**Table 1.** Characteristics of study participants with eye symptoms.

Characteristics		Participants with eye symptoms reported within the past 2 wk (N=27,336), n (%)
**Sex**		
	Female	19,323 (70.7)
	Male	8014 (29.3)
**Age group**		
	Young adults	11,118 (40.7)
	Middle-aged	5063 (18.5)
	Older adults	11,155 (40.8)
**Region**		
	North	3084 (11.3)
	Central	11,874 (43.4)
	South	12,378 (45.3)
**Chronic diseases**		
	Yes	2508 (9.2)
	No	24,829 (90.8)
**Residence**		
	Urban	4135 (15.1)
	Rural	23,301 (84.9)
**Marital status**		
	Married	14,140 (51.7)
	Separated/divorced	2409 (8.8)
	Widower/widowed	5948 (21.8)
	Not married	4841 (17.7)
**Education**		
	None	21,032 (76.9)
	Primary	1475 (5.4)
	Secondary	4829 (17.7)

### Factors Associated With ECU

The *χ*^2^ test was run as part of the bivariate analysis to assess the association between ECU and various demographic factors. The test showed that a higher proportion of ECU was associated with males than females such that 68.2% (5467/8014) of males sought eye care services compared to 57.4% (11,097/19,322) of females. The percentage of participants who sought care increased with a higher level of education, as depicted by 53.6% (11,283/21,033) of participants without education who sought care, while 79.1% (1166/1475) of participants with primary education and over 85% (4115/4828) of participants with secondary school education (*P*<.001) used eye care services. Pearson *χ*^2^ showed that the difference was statistically significant (*P*<.001). According to place of residence, 66.1% (2733/4134) of urban residents sought care compared to 59.6% (13,831/23,202) of rural residents. The difference was statistically different (*P*<.001). According to the presence of chronic illness, 77.7% (1949/2508) of participants who had a chronic condition sought care while 88.2% (14,616/24,829) of those participants with no chronic illness sought care (*P*<.001). Based on the region of residence, 42.7% (1318/3084), 50.4% (5992/11,875), and 74.8% (9254/12,378) of the participants sought care from the north, central, and southern regions, respectively (*P*<.001). Considering marital status, 13.8% (2286/4840) of participants who were not married sought care, while 61.8% (10,239/14,140) of married people sought care. Among those divorced, only 40.1% (968/2409) of participants sought care and 18.5% (3072/5948) of those who sought care were widowers/widows (*P*<.001). Regarding age, 54.9% (6110/11,118) of young adults sought care, while 90.2% (4569/5063) of middle-aged adults and 52.7% (5885/11,155) of older adults used eye care services (*P*<.001).

### Places Where Participants Sought Help

A total of 16,564 (60.6%, 95% CI 60.0%-61.2%) of the 27,336 participants sought eye care from a medical/health facility. Of the 16,564 participants who sought care from a health facility, 14,173 (85.6%) visited a government facility, and 463 (2.7%) obtained drugs from their local pharmacy. Among those who did not seek care, 2950 of 6343 (46.5%) attributed it to lack of funds, while 3393 of 6343 (53.5%) did not think it was a serious illness (Table 2).[Table T2][Table T2 T2][Table T2 T2 T2]

**Table 2. T2:** Distribution of actions taken by participants with eye symptoms 2 weeks before the survey.

			Participants (N=27,366), n (%)
**Did not seek health care^a^**			6343 (23.1)
	Did nothing, not a serious illness		3393 (53.4)
	Did nothing, no money		2950 (46.8)
**Sought health care**			
	**Care at health facility^b^**		16,565 (60.5)
		Government	14,173 (85.5)
		Private	1316 (7.9)
		Church/mission	613 (3.7)
		Pharmacy store	463 (2.7)
	**Care not at health facility^a^**		3788 (13.8)
		Personal remedies	2057 (54.3)
		Grocery store	1123 (29.6)
		Traditional healer	608 (16.2)

a These actions do not constitute eye care use.

b These actions constitute eye care use.

### Factors Affecting ECU

A logistic regression was performed to ascertain the effects of residence, region, education qualification, chronic illness, sex, and age on the likelihood that participants reported ECU. The logistic regression model was statistically significant (*χ*^2^_8_=27.4; *P*<.001). The model explained 34.0% (Nagelkerke *R*^2^) of the variance in ECU. Males were more likely to report ECU than females (odds ratio [OR] 1.2, 95% CI 1.104-1.290). Having a chronic condition was associated with a reduction in the likelihood of ECU (OR 0.6, 95% CI 0.547-0.708). Residents in the urban area were 1.2 times more likely to exhibit ECU than residents in rural areas (OR 1.2, 95% CI 1.118-1.375). Those with a higher education qualification were 6 times more likely to seek eye care at a medical facility than those without a formal education (OR 6.6, 95% CI 5.927-7.366). Middle-aged participants were 7 times more likely to use eye care services than young adults (OR 7, 95% CI 6.782-8.476), while older adults were less likely to use eye care than middle-aged adults.

## Discussion

The prevalence of ECU in our study was similar to a previous report [11]. However, other authors report comparatively lower rates of ECU [6,15]. On the contrary, others reported larger ECU rates [16,24]. There is a discrepancy in the rate of ECU as it varies widely ranging from 18% to 82% due to study settings, sample size, and study population [6]. In part, the variation could be due to different operational definitions of ECU. For instance, we defined ECU as seeking care for ocular problems at a medical facility 2 weeks before the study, whereas the American Optometric Association defines ECU as the use of eye care services in the preceding 3 years [25]. The high rate of ECU in our study could be attributed to the affordability of eye care services in the country [26].

The majority of participants who sought care in our study visited government hospitals. On the contrary, Kyaw et al [27] found that the most frequently visited place is private hospitals, and Morka et al [6] reported that the most preferred place of choice is an eye center [28]. The results vary depending on the study setting. The results of our study are not surprising considering that the government is the chief actor in health service delivery in the country [26].

Regarding the reasons for not seeking care, we found that the majority of participants cited a lack of funds. Cost is a common barrier in many low-income nations [29,30]. Although eye care is provided free of charge in Malawi, individuals still incur pocket expenditures in the form of transport costs [31]. Assessing barriers to ECU is beyond the scope of this paper; nevertheless, our finding endorses the need to scale up outreach programs to mitigate costs incurred when reaching a health facility.

Another larger group of people did not seek care for their eye problems due to “negligence” similar to the previous report [30]. This reflects poor-functioning eye health awareness programs and could be a contributing factor to the late presentation of ocular conditions to the hospital. Therefore, the findings of this study suggest that community sensitization and eye health education programs emphasize the gravity of eye problems and the significance of early presentation to the hospital.

A previous report noted that the prevalence of self-medication was 40% [32]; on the contrary, this study found that a decreased number of participants resorted to self-medication. The difference could be due to different sample sizes. Our data used a nationwide sample, unlike the previous study that recruited in only two districts. Regardless, self-medication is a custom in Africa where the majority of eye problems do not go beyond unorthodox alternatives [32]. Our study underscores the significance of incorporating nonorthodox approaches into the national eye health system.

Concerning age, ECU was highest among young adults but dropped among older adults despite a high prevalence of eye symptoms. The trend is similar to Zhao and colleagues [33]; however, it is in disagreement with others [6,34]. In general, major causes of eye diseases are age related; thus, demand for eye services increases with age [6]. The results of our study could be attributed to a lack of social support. Research suggests decreased ECU among older adults due to the lack of an escort to the health facility [6]. This highlights an inefficient eye care delivery system with top-heavy needs and supply. Hence, we recommend gearing services toward older adults, such as the aforementioned vision-screening programs oriented toward senior citizens.

Arguably, one’s cultural values have an impact on access to health. Our study has shown that male subjects are associated with ECU more than females, similar to previous authors [35,36]. In contrast, others report females’ predisposition to ECU [33,34]. Nonetheless, Akuwoah and colleagues [29] found no statistically significant difference between genders. The variation can be explained by different cultural backgrounds in different populations. The high ECU rate among males in our study can be explained by the Malawian cultural scene. Researchers demonstrated that Malawian women’s underuse of necessary health care was a factor in decision-making and attitude [8]. We advocate for women’s empowerment and mitigation of gender stereotypes to improve equal access to eye care services in the country. The literature suggests that education is instrumental in curbing such gender disparities [8].

Generally, education is a key affluence factor in health as it directly relates to awareness [37]. Our investigation has shown that the rate of ECU is higher among those with more education similar to previous studies [34,37]. This provides an attractive target for strategies to improve ECU through measures to keep citizens in school, promote equal education opportunities, and design eye health messages targeting citizens who are illiterate.

Surprisingly, this review has shown that having a chronic disease is negatively associated with ECU. However, other researchers [16,34] found that the absence of diabetes was associated with low ECU. Etiologically, most ocular conditions are caused by chronic diseases [11]. Given that the study was cross-sectional and that participants had just 2 weeks to report eye issues, it is crucial to note that chronic illnesses could be more common than reported in this study. Moreover, we attributed the results of our study to a lack of knowledge and awareness. A recent study found that understanding health and disease is a key determinant of seeking health care among persons with chronic conditions in Malawi [38]. When health education messages demonstrate that there is a potential health risk and convince people that certain behaviors can prevent such risks, the likelihood of change is increased substantially [10]. An imperative but unanswered question is how individual chronic diseases affect ECU. Regardless, our findings echo the significance of awareness campaigns among people with chronic diseases.

Our investigation revealed that people from urban areas use eye care services more than their rural counterparts comparable to previous studies [24]. The rural-urban disparity can be explained by less available ophthalmic human resources in rural Malawi. For instance, 11 of 12 ophthalmologists in the country are based in urban areas [39]. The results of the study suggest inequity in deployment policies; hence, task-shifting approaches would ensure coverage in hard-to-reach areas.

Concerning region of origin, ECU in Malawi varies, with the south registering the highest and the north having the lowest levels of ECU. We cannot explain the regional variation in our study.

A particular strength of this study is the use of a countrywide population-based data set that provides ample sample size and statistical power to reconnoiter the association between ECU and its associated factors. Nevertheless, our study is not without drawbacks. First, the study is based on subjective responses that are prone to recall bias by the study participants. Another important caveat of our study is that we did not include the association between economic status and ECU including wealth and out-of-pocket expenditure. In addition, we did not evaluate realized access, patient satisfaction, and quality of care due to a lack of objective measures.

In summary, we report a novel study on the rate of ECU and its associated demographic factors among a population of Malawian adults using data from a national survey. The results demonstrate that in Malawi access to eye care services is entrenched in social disadvantages. ECU is low among older adults due to a lack of social support. Unequal distribution of resources between urban and rural areas could cause disparities in ECUs. There were regional variations in the use of eye services. Measures and interventions to improve ECU should target strategies to increase education opportunities for all, women empowerment, and outreach programs for rural residents and older adults, including task-shifting programs.

## Abbreviations

ECU: eye care use
HSB: health-seeking behavior
IHS: Integrated Health Survey
OR: odds ratio
